# Familial adenomatous polyposis is associated with a marked decrease in alkaline sphingomyelinase activity: a key factor to the unrestrained cell proliferation?

**DOI:** 10.1038/sj.bjc.6690682

**Published:** 1999-09

**Authors:** E Hertervig, Å Nilsson, J Björk, R Hultkrantz, R-D Duan

**Affiliations:** Departments of Medicine, Cell Biology, Lund University Hospital, Lund, 221 85, Sweden; Department of Gastroenterology and Hepatology, Karolinska Hospital, Stockholm, 171 76, Sweden

**Keywords:** alkaline sphingomyelinase, FAP, adenoma, tumorigenesis, human

## Abstract

The hydrolysis of sphingomyelin generates key molecules regulating cell growth and inducing apoptosis. Data from animal cancer models support an inhibitory role for this pathway in the malignant transformation of the colonic mucosa. In the intestinal tract, a sphingomyelinase with an optimum alkaline pH has been identified. We recently found that the activity of alkaline sphingomyelinase is significantly decreased in colorectal adenocarcinomas, indicating a potential anticarcinogenic role of this enzyme. To further examine whether the reduction of sphingomyelinase is present already in the premalignant state of neoplastic transformation, we measured sphingomyelinase activities in patients with familial adenomatous polyposis (FAP) and in sporadic colorectal tubulovillous adenomas. Tissue samples were taken from adenomas and surrounding macroscopically normal mucosa from 11 FAP patients operated with ileorectal anastomosis, from three FAP patients with intact colon, from 13 patients with sporadic colorectal adenomas and from 12 controls. Activities of acid, neutral and alkaline sphingomyelinase were measured together with alkaline phosphatase. In FAP adenoma tissue, alkaline sphingomyelinase activity was reduced by 90% compared to controls (*P* < 0.0001), acid sphingomyelinase by 66% (*P* < 0.01) and neutral sphingomyelinase by 54% (*P* < 0.05). Similar reductions were found in the surrounding mucosa. In sporadic adenoma tissue, only alkaline sphingomyelinase was reduced significantly, by 57% (*P* < 0.05). Alkaline phosphatase was not changed in FAP adenomas, but decreased in the sporadic adenomas. We conclude that the markedly reduced levels of alkaline sphingomyelinase activities in FAP adenomas and in the surrounding mucosa may be a pathogenic factor that can lead to unrestrained cell proliferation and neoplastic transformation. © 1999 Cancer Research Campaign


*British Journal of Cancer* (1999) **81**(2), 232–236
© 1999 Cancer Research Campaign
Article no. bjoc.1999.0682

### Familial adenomatous polyposis is associated with a marked decrease in alkaline sphingomyelinase activity: a key factor to the unrestrained cell proliferation?

**E Hertervig[1], Å Nilsson[1], J Björk[3], R Hultkrantz[3] and R-D Duan[2]**

Departments of [1]Medicine and [2]Cell Biology, Lund University Hospital, 221 85 Lund, Sweden; [3]Department of Gastroenterology and Hepatology, Karolinska Hospital, 171 76 Stockholm, Sweden

**Summary** The hydrolysis of sphingomyelin generates key molecules regulating cell growth and inducing apoptosis. Data from animal cancer models support an inhibitory role for this pathway in the malignant transformation of the colonic mucosa. In the intestinal tract, a sphingomyelinase with an optimum alkaline pH has been identified. We recently found that the activity of alkaline sphingomyelinase is significantly decreased in colorectal adenocarcinomas, indicating a potential anticarcinogenic role of this enzyme. To further examine whether the reduction of sphingomyelinase is present already in the premalignant state of neoplastic transformation, we measured sphingomyelinase activities in patients with familial adenomatous polyposis (FAP) and in sporadic colorectal tubulovillous adenomas. Tissue samples were taken from adenomas and surrounding macroscopically normal mucosa from 11 FAP patients operated with ileorectal anastomosis, from three FAP patients with intact colon, from 13 patients with sporadic colorectal adenomas and from 12 controls. Activities of acid, neutral and alkaline sphingomyelinase were measured together with alkaline phosphatase. In FAP adenoma tissue, alkaline sphingomyelinase activity was reduced by 90% compared to controls ($P < 0.0001$), acid sphingomyelinase by 66% ($P < 0.01$) and neutral sphingomyelinase by 54% ($P < 0.05$). Similar reductions were found in the surrounding mucosa. In sporadic adenoma tissue, only alkaline sphingomyelinase was reduced significantly, by 57% ($P < 0.05$). Alkaline phosphatase was not changed in FAP adenomas, but decreased in the sporadic adenomas. We conclude that the markedly reduced levels of alkaline sphingomyelinase activities in FAP adenomas and in the surrounding mucosa may be a pathogenic factor that can lead to unrestrained cell proliferation and neoplastic transformation.

© 1999 Cancer Research Campaign

**Keywords:** alkaline sphingomyelinase; FAP; adenoma; tumorigenesis; human

The hydrolytic products of sphingomyelin (SM) triggered by sphingomyelinases (SMase) are important molecules that regulate cell proliferation, differentiation and programmed cell death (Kolesnik, 1991; Hannun and Linnardi, 1993). Three types of SMase have been identified so far. The first two, termed acid and neutral SMase respectively, have been found in many tissues and are considered as common cellular enzymes (Chatterjee, 1994; Spence, 1994). The third enzyme, alkaline SMase, has been located specifically to the intestinal tract, where high levels are found in the small intestine and lower in the colon with a gradual decline towards rectum (Nilsson, 1969; Duan et al, 1995*a*, 1995*b*). This enzyme may play an important role in the digestion of dietary SM (Nyberg et al, 1997) and provide the intestinal mucosa with ceramide, a key molecule to induce apoptosis (Obeid et al, 1993).

Recent animal studies indicate that the digestion and hydrolysis of SM may have an inhibitory effect on neoplastic transformation in the colorectal mucosa. Human colonic carcinomas, as well as the colonic mucosa of rats treated with colonic carcinogens, are associated with an accumulation of SM (Dudeja et al, 1986; Merchant et al, 1995). In normal mice, dietary supplement of SM has been shown to reduce the number of aberrant colonic crypt foci (considered to be early biomarkers of dysplastic change in the epithelium) and mice given 1,2-dimethyl-hydrazine, a colonic carcinogen, together with SM, showed an increase in the proportion of adenomas versus adenocarcinomas, indicating a possible chemopreventive role (Dillehay et al, 1994; Schmelz et al, 1996). We recently found that human colorectal carcinomas had a 75% reduction of alkaline SMase activity compared to the normal adjacent mucosa (Hertervig et al, 1997).

Familial adenomatous polyposis (FAP) is an autosomal dominant disease that affects 1 in 7000 individuals. Patients with FAP typically develop hundreds to thousands of colorectal adenomatous polyps, and the large numbers of polyps virtually guarantee that some of the polyps in each affected individual will progress to invasive carcinoma. The critical underlying mechanism for tumour initation is the germline mutation of the adenomatous polyposis (APC) gene; however, a progress to invasive carcinoma requires a series of other somatic mutations including the p53 gene, the k-*ras* gene and the ‘deleted in colorectal cancer’ gene (DCC) (Kinzler, 1994).

In order to find out whether the decrease in alkaline SMase activity is an early phenomenon in the colorectal tumorigenesis, occurring before malignant transformation, we extended our previous study by measuring alkaline SMase activity in colorectal mucosa of FAP patients as well as in sporadic adenomas. A sharp and specific decrease of alkaline SMase activity in both adenomas and surrounding mucosa of FAP patients has been demonstrated, which indicates that this enzyme may be an important factor in the unrestrained cell proliferation and malignant transformation in the colorectal mucosa.

*Received 4 September 1998*
*Revised 15 February 1999*
*Accepted 30 March 1999*

*Correspondence to:* E Hertervig

### MATERIALS AND METHODS

### Materials

Biopsies were obtained from rectal adenomas in 11 FAP patients operated with colectomy and ileorectal anastomosis (mean age 49.5 years, range 30–73 years; five males, six females). Six of the patients also had biopsies taken from macroscopically normal surrounding mucosa. In addition, rectal biopsies were obtained from adenomas and the surrounding mucosa in three other FAP patients with intact colon (two males, 23 and 27 years; one female, 22 years).

Sporadic polyps were obtained from the rectosigmoid colon by colonoscopy and polypectomy in 15 patients. Two of the polyps that had carcinoma on histopathological examination were excluded. The remaining 13 polyps were benign colorectal tubulovillous adenomas with varying dysplasia (mean age 72 years, range 49–82 years; seven males, six females). The adenomas are characterized in Table 1. Biopsies were also taken from macroscopically normal adjacent mucosa. Control rectal biopsies were obtained from 12 patients referred to colonoscopy for abdominal symptoms, but with a macroscopically normal colonoscopy and without microscopical pathological findings (mean age 45.7 years, range 22–83 years; four males, eight females). Permission to obtain biopsies were obtained by the local ethical committees in Lund and Stockholm.

Purified milk SM (purity > 98%) and choline labelled $^{14}$C-SM (56 µCi mg$^{-1}$) were kindly provided by Lena Nyberg, Swedish Dairy Association and by Peter Ström, Astra Draco, Lund (Stoffel, 1975; Nyberg et al, 1996). Taurocholate (TC), taurodeoxycholate (TDC), glycocholate (GC), glycochenodeoxycholate (GCDC), benzamidine and phenylmethylsulphonyl fluoride (PMSF) were purchased from Sigma Co (St Louis, MO, USA).

### Sample preparation

The biopsy samples were homogenized in 0.5 ml 0.25 M sucrose buffer containing 5 mM magnesium chloride ($MgCl_2$), 0.15 M potassium chloride (KCl), 50 mM $KH_2PO_4$, 1 mM PMSF, 1 mM benzamidine and 10 mM TC, pH 7.4, followed by sonication for 10 s. After centrifugation at 10 000 rpm at 4°C for 15 min, the supernatant was saved for determination of SMase activity, alkaline phosphatase activity and protein content.

### Sphingomyelinase activity assay

The activity of alkaline SMase was determined according to Duan et al (1995*a*, 1995*b*) which is a modification of an original method for neutral SMase (Gatt, 1975). Briefly, samples were added in 375 µl Tris–EDTA buffer pH 9.0 to a final volume of 0.4 ml, containing 50 mM Tris, 0.15 M sodium chloride (NaCl), 2 mM EDTA and 3 mM bile salt mixture with a molar ratio TC:TDC:GC:GCDC being 3:2:1.8:1. Such a bile salt mixture had previously shown to have the maximum stimulatory effect on alkaline SMase (Duan 1995*a*, Nyberg et al, 1996). The supplement of EDTA in the buffer served to inhibit the neutral SMase activity which is magnesium ion-dependent with a pH optimum at 7.5 (Chatterjee, 1994). $^{14}$C-SM dissolved in ethanol was suspended in 0.9% NaCl containing 3 mM bile salt mixture. The reaction was started by adding 100 µl $^{14}$C-SM (40 000 dpm) suspension, incubated at 37°C for 30 min, and terminated by adding 2 ml chloroform:methanol (2:1). After phase partition and centrifugation, an aliquot of the upper phase containing the cleaved phosphocholine was taken and the radioactivity was counted by liquid scintillation. The activity was calculated and normalized as nmol h$^{-1}$ mg$^{-1}$ sample protein.

The activities of neutral and acid SMases were measured by the same procedure described above except small modifications of the buffers. Neutral SMase was assayed in buffer containing 50 mM Tris, 0.15 M NaCl, 2 mM $MgCl_2$ and 3 mM bile salt mixture pH 7.5, whereas acid SMase was determined in 50 mM Tris–maleate buffer containing 0.15 M NaCl and 3 mM bile salt mixture pH 5.0 (Duan et al, 1995*b*).

**Table 1** Characteristics of endoscopically removed sporadic rectosigmoid colorectal tubulovillous adenomas from 13 patients

| Patient no. | Sex | Age | Dysplasia | Size (mm) |
|---|---|---|---|---|
| 1 | Female | 78 | Moderate | 10 |
| 2 | Female | 49 | Mild | 5 |
| 3 | Female | 72 | Mild | 5 |
| 4 | Male | 82 | Severe | 25 |
| 5 | Male | 75 | Moderate | 10 |
| 6 | Female | 80 | Moderate | 8 |
| 7 | Male | 63 | Mild | 5 |
| 8 | Male | 72 | Moderate | 14 |
| 9 | Male | 67 | Moderate | 6 |
| 10 | Male | 71 | Severe | 5 |
| 11 | Female | 76 | Mild | 9 |
| 12 | Male | 82 | Severe | 20 |
| 13 | Female | 69 | Severe | 8 |

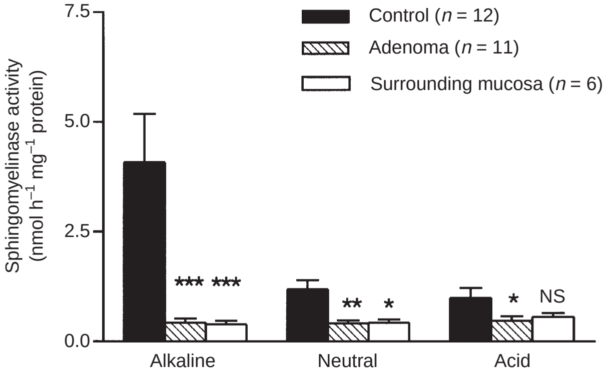

**Figure 1** Alkaline, neutral and acid SMase activities (mean ± s.e.m.) in rectal biopsies from normal mucosa of a control group, from FAP adenomas and from the surrounding mucosa in the FAP patients. The samples were homogenized followed by sonication. After centrifugation, the SMase activities and protein content in the supernatant were determined. *$P$ < 0.05, **$P$ < 0.01, ***$P$ < 0.001, NS = no significance

### Other biochemical determinations

Protein content was assayed by a kit obtained from Bio-Rad Co. (Hercules, CA, USA), using bovine serum albumin as a standard. A kit obtained from Sigma using p-nitrophenyl phosphate as a substrate was used to assay alkaline phosphatase.

### Statistical analysis

The data were presented as mean ± standard error of mean (s.e.m.) and the significant differences ($P$ < 0.05) were evaluated using the

*© 1999 Cancer Research Campaign*

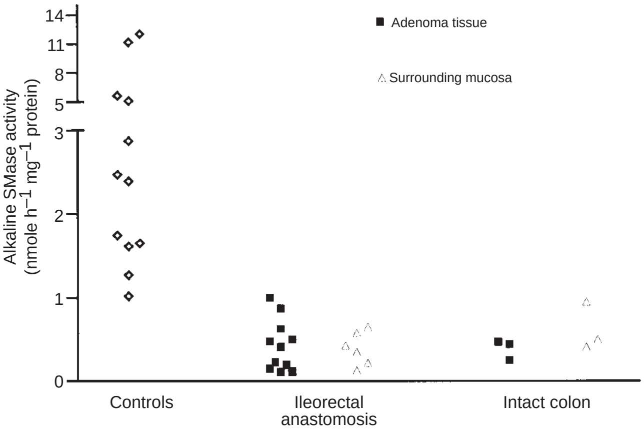

**Figure 2** Comparison of alkaline SMase activities in rectal biopsies from FAP patients, with an ileorectal anastomosis to those with an intact colon and a control group. Biopsies were taken both from adenoma tissue and the surrounding mucosa

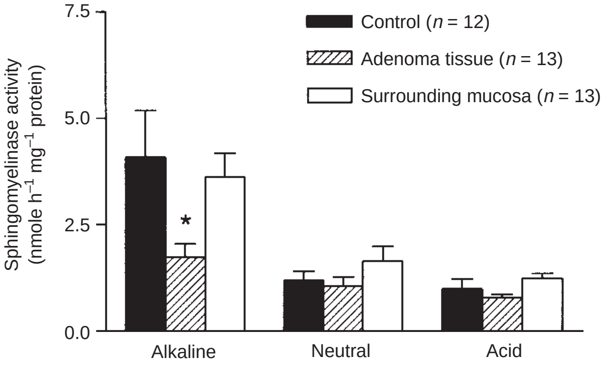

**Figure 3** Alkaline, neutral and acid SMase activities (mean ± s.e.m.) in sporadic adenomas and the normal surrounding mucosa in the rectosigmoid colon, compared to a control group. $^*P < 0.05$

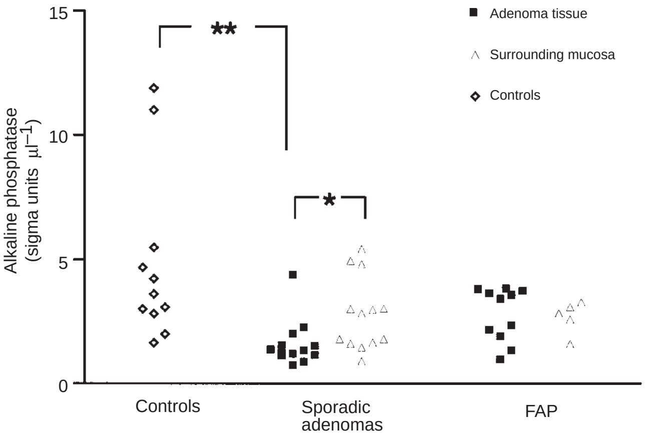

**Figure 4** Alkaline phosphatase activities in adenoma tissue and in the surrounding normal mucosa from patients with FAP adenomas and sporadic adenomas together with a control group

Mann–Whitney *U*-test, except for the paired data where Wilcoxon signed rank test was used.

### RESULTS

### Changes in SMase activities in FAP-adenomas

We first studied the activities of all three types of SMases in rectal adenoma tissue and normal surrounding mucosa in 11 FAP patients who had been operated with an ileorectal anastomosis and compared them to the activities of a control group with macro- and microscopically normal rectal mucosa. As shown in Figure 1, alkaline SMase activity in adenoma tissue was markedly reduced by 90% compared to controls ($P < 0.0001$). Neutral and acid SMase activities were, to a lesser extent, also decreased by 66% ($P = 0.0028$) and 54% ($P = 0.021$) respectively. Similar reductions of SMase activities were also found in the surrounding normal mucosa of the FAP patients, by 90% for alkaline SMase ($P = 0.0009$), by 64% for neutral SMase ($P = 0.022$) and by 43% for acid SMase (NS).

Since the data above were obtained from patients who had undergone ileorectal anastomosis, the possible influence of the operation on alkaline SMase activity needed to be examined. We therefore further assayed the enzyme activity in rectal biopsies from three young FAP patients with intact colon. The results are shown in Figure 2. Alkaline SMase activities in the rectum of non-operated patients, both concerning adenoma tissue and the adjacent mucosa, did not differ from those in the operated group, indicating that the operation did not affect the outcome.

### Changes in SMase activities in sporadic adenomas

When the SMase activities were determined in sporadic adenomatous tissue from the rectosigmoid region, we found that the activity of alkaline SMase was reduced by 57% compared to controls ($P = 0.014$), whereas acid and neutral SMase did not differ significantly. The adjacent mucosa did not differ significantly from controls in any of the SMases (Figure 3). The magnitude of the decrease of alkaline SMase activity did not correlate with the size of the adenoma or with the grade of dysplasia.

### Changes in alkaline phosphatase activity

For comparison, alkaline phosphatase, which, similarly to alkaline SMase, is located in the brushborder, was determined. There were no significant differences of the enzyme activity between the adenoma tissue and the surrounding mucosa in FAP patients, and no difference could be found comparing any of the values to controls. In sporadic adenoma tissue, alkaline phosphatase activity was reduced by 67% compared to controls ($P = 0.0061$) and by 43% compared to the surrounding mucosa in the same patients ($P = 0.040$) (Figure 4). However, there was no significant correlation between alkaline SMase and alkaline phosphatase activities (Figure 5).

### DISCUSSION

SMase, by catalyzing the hydrolysis of SM, generates multiple molecules, some of which have been considered as tumour suppressors due to their antiproliferative effects (Hannun and Linnardic, 1993). We recently showed that in the tissue of human colorectal carcinoma alkaline SMase activity was decreased by

*© 1999 Cancer Research Campaign*

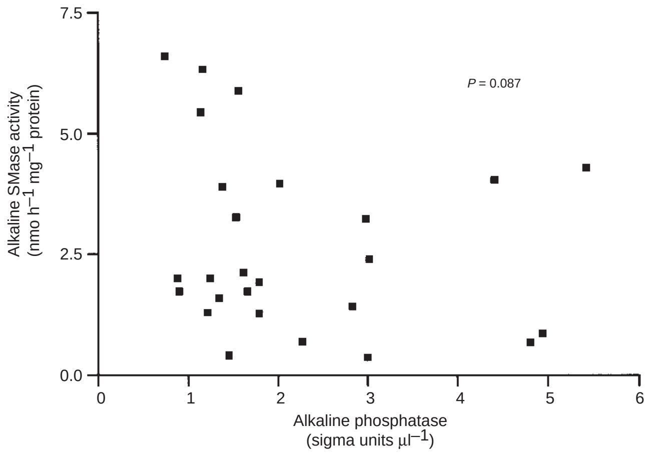

**Figure 5** The correlation between alkaline phosphatase and alkaline SMase activities in biopsies from patients with sporadic colorectal adenomas

75% as compared with the surrounding mucosa, implying a potential role of this enzyme in colonic tumorigenesis (Hertervig et al, 1997). In this study, we extended our previous studies by demonstrating a marked decrease by 90% of alkaline SMase activity in the colorectal adenomas and in the surrounding mucosa of FAP patients compared to controls. The reduction of alkaline SMase activity is specific, not only because of the predominant decrease as compared to acid and neutral SMase, but also because there was no change of alkaline phosphatase activity, a brush border enzyme similar to alkaline SMase. In sporadic adenomas, alkaline phosphatase activity was reduced but we found no correlation of the reduction to changes of alkaline SMase. Although most of the data from FAP patients were obtained from patients who had undergone colectomy and ileorectal anastomosis, the operation is not attributable to the decrease of alkaline SMase activity, since low levels of the enzyme activities were similarly found in the non-operated FAP patients.

An important question is whether a difference in the amounts of tissue components between normal mucosal and adenoma may influence the results significantly. This is unlikely for the following reasons. First, the reduction of alkaline SMAse was seen also in the normal appearing mucosa of FAP patients and histological features of randomly taken colonoscopic biopsies from normal appearing mucosa of FAP patients rarely show any adenomatous epithelial proliferation (Yang et al, 1998). Secondly, the similar values per mg protein of another brush border enzyme, alkaline phosphatase support that the reduction of alkaline SMase is specific. In our view, tissue heterogeneity could not explain any significant part of the 90% decrease in alkaline SMase in the normal appearing mucosa from FAP patients as compared with controls. Finally, concerning the adenomas, it is generally recognized that colonoscopic biopsies from benign adenomas contain very high proportions of mucosal cells (approximately 90%) and exhibit little variations in this respect. Thus, when comparing samples from adenomas and normal mucosa the mucosal content of brush border enzymes in adenomas may be somewhat overestimated. In this study the effect would be to diminish rather than to enhance the difference between normal mucosa and adenomas.

As opposed to our previous study on non-inherited colorectal carcinoma, where the reduction of alkaline SMase activity only occurred in the carcinoma tissue (Hertervig et al, 1997), the present study on FAP patients demonstrates decreased alkaline SMase activity in both adenoma tissue and in the surrounding normal-appearing mucosa. This may indicate that the reduction is an early phenomenon and may have genetic connections with the disease. It is well known that FAP is an inherited autosomal dominant disorder and the consequence of a germline mutation of the adenomatous polyposis coli (APC) gene (Groden et al, 1991; Kinzler et al, 1991). The APC gene has been proposed as ‘the gatekeeper gene’ in the colorectal mucosa, and has recently been suggested to be implicated in cell signal transduction affecting processes like cell growth inhibition (Baeg et al, 1995), apoptosis (Browne et al, 1994) and migration (Wong et al, 1996). Studies in humans with FAP, as well as in mice with analogous mutations, have suggested that the rate-limiting step in the tumour initiation is a somatic mutation of the wild-type APC allele inherited from the unaffected parent (Icii et al, 1992; Luongo et al, 1994). APC mutations have also been found in humans in the earliest neoplastic lesions, the dysplastic aberrant crypt foci (Jen et al, 1994). The APC protein interacts with at least six proteins, among them β-catenin (Su, 1994). Just recently β-catenin has been shown to function as a transcriptional activator when complexed with members of the Tcf family of DNA binding proteins and wild-type APC has the ability to suppress signalling by the β-catenin-Tcf complex (Korinek et al, 1997). Furthermore, mutant APC genes are defective in their ability to down-regulate β-catenin-mediated transcriptional activity (Morin et al, 1997). Missing at present is the knowledge of which genes are activated by the β-catenin-Tcf complex. SMase is a key enzyme to trigger a signal transduction pathway leading to inhibition of cell proliferation and to apoptosis. Thus, in view of the present data and our previous work on colorectal carcinoma (Hertervig et al, 1997), it is tempting to speculate about a possible connection between the APC protein and the regulation of alkaline SMase expression.

During the last 5 years, the possible link between hydrolysis of SM and colonic tumorigenesis has become a focus for investigation. It has been shown that supplement of SM in the diet to normal mice significantly reduced the number of aberrant crypt foci in the colon and inhibited the development of colon carcinoma induced by chemical carcinogens (Dillehay et al, 1994; Schmelz et al, 1996). In agreement with this, human colonic carcinomas as well as colonic mucosa of rats treated with chemical dimethylhydrazine, show an accumulation of SM (Dudeja et al, 1986; Merchant et al, 1995), which may well be the result of a reduced SMase activity as shown in our previous work (Hertervig et al, 1997). In addition, ursodeoxycholate, a bile salt that has been shown to inhibit experimental colon cancer development (Earnest et al, 1994) has recently been found to increase alkaline SMase activity in rats (Duan et al, 1998).

In summary, this study shows that a very low level of alkaline SMase is a feature of both adenoma tissue and the macroscopically normal appearing mucosa in FAP patients. and thus it may represent a marker of increased proliferative potential in the mucosa, or even further, be an important pathogenic mechanism behind the unrestrained cell proliferation seen in the mucosa of FAP patients.

### REFERENCES

Baeg G-H, Matsumine A, Kuroda T et al. (1995) The tumour suppressor gene product APC blocks cell cycle progression from G0/G1 to S phase. *EMBO J* **14**: 5618–5625

© 1999 Cancer Research Campaign

Browne SJ, Williams AC, Hague A et al. (1994) Loss of APC protein expressed by human colonic epithelial cells and the appearance of a specific low-molecular-weight form is associated with apoptosis in vitro. *Int J Cancer* **59**: 59–64
Chatterjee S (1994) Neutral sphingomyelinase. *Adv Lip Res* **26**: 25–57
Dillehay DL, Sebb SK, Schmelz E-M et al. (1994) Dietary sphingomyelin inhibits 1,2-dimethylhydrazine-induced colon cancer in CF-1 mice. *J Nutr* **124**: 615–620
Duan R-D, Nyberg L and Nilsson Å (1995*a*) Alkaline sphingomyelinase activity in rat gastro-intestinal tract: distribution and characteristics. *Biochim Biophys Acta* **1259**: 49–55.
Duan R-D, Hertervig E, Nyberg L et al. (1995*b*) The distribution of alkaline sphingomyelinase activity in human beings and animals: tissue and species differences. *Dig Dis Sci* **41**: 1801–1806
Duan R-D, Cheng Y, Tauschel H-D et al. (1998) Effects of ursodexycholate and other bile salts on levels of rat intestinal sphingomyelinase: a potential implication for tumorigenesis. *Dig Dis Sci* **43**: 226–232
Dudeja PK, Dahiya R, Brasitus TA et al. (1986) The role of sphingomyelin synthetase and sphingomyelinase in 1,2-dimethylhydrazine-induced lipid alterations of rat colonic plasma membranes. *Biochim Biophys Acta* **863**: 309–312
Earnest DL, Holubec H, Wali RK et al. (1994) Chemoprevention of azoxymethane-induced colonic carcinogenesis by supplemental dietary ursodeoxycholic acid. *Cancer Res* **54**: 5071–5074
Gatt S (1975) Magnesium-dependent sphingomyelinase. *Biochim Biophys Acta* **68**: 235–241
Groden J, Thliveris A, Samowitz W et al. (1991) Identification and characterization of the familial adenomatous polyposis gene. *Cell* **66**: 589–600
Hannun YA and Linnardic CM (1993) Sphingolipid breakdown products: anti-proliferative and tumour-suppressor lipids. *Biochim Biophys Acta* **1154**: 223–236
Hertervig E, Nilsson Å, Nyberg L et al. (1997) Alkaline sphingomyelinase is decreased in human colorectal carcinoma. *Cancer* **79**: 448–453
Icii S, Nakatsuru S, Furuyama J et al. (1992) Inactivation of both APC alleles in an early stage of colonic adenomas in a patient with familial adenomatous polyposis (FAP). *Hum Mol Genet* **1**: 387–390
Jen J, Powell SM, Papadopoulos N et al. (1994) Molecular determinants of dysplasia in colorectal lesions. *Cancer Res* **54**: 5523–5526
Kinzler KW and Vogelstein B (1996) Lessons from hereditary cancer. *Cell* **87**: 159–170
Kinzler KW, Nilbert MC, Su L-K et al. (1991) Identification of FAP locus genes from chromosome 5q21. *Science* **253**: 661–665
Kolesnik RN (1991) Sphingomyelin and derivatives as cellular signals. *Prog Lipid Res* **30**: 1–38
Korinek V, Barker N, Morin PJ et al. (1997) Constitutive transcriptional activation by a β-catenin-Tcf complex in $APC^{-/-}$ colon carcinoma. *Science* **275**: 1784–1787
Luongo C, Moser AR, Gledhill S et al. (1994) Loss of Apc+ in intestinal adenomas from Min mice. *Cancer Res* **54**: 5947–5952
Merchant TE, Diamantis PM, Lauwersa G et al. (1995) Characterization of malignant colon tumours with 31P nuclear magnetic resonance phospholipid and phosphatic metabolite profiles. *Cancer* **76**: 1715–1723
Morin PJ, Sparks AB, Korinek V et al. (1997) Activation of β-catenin-Tcf signaling in colon cancer by mutations in β-catenin or APC. *Science* **275**: 1787–1790
Nilsson Å (1969) The presence of sphingomyelin- and ceramide-cleaving enzymes in the small intestinal tract. *Biochim Biophys Acta* **176**: 339–347
Nyberg L, Duan R-D, Axelson J et al. (1996) Identification of an alkaline sphingomyelinase in human bile. *Biochim Biophys Acta* **1300**: 42–48
Nyberg L, Nilsson Å, Lungren P et al. (1997) Localization and capacity of sphingomyelin digestion in the rat intestinal tract. **8**: 112–117
Obeid LM, Linardic CM, Karoloak LA et al. (1993) Programmed cell death induced by ceramide. *Science* **259**: 1769–1771
Schmelz EM, Dillehay DL, Webb SK et al. (1996) Sphingomyelin consumption suppresses abberant crypt foci and increases the proportion of adenomas versus adenocarcinomas in CF1 mice treated with 1,2-dimethylhydrazine: implications for dietary sphingolipids and colon carcinogenesis. *Cancer Res* **56**: 4936–4941
Spence MW (1994) Sphingomyelinases. *Adv Lip Res* **26**: 3–23
Stoffel W (1975) Chemical synthesis of choline-labeled lecithins and sphingomyelins. *Methods Enzymol* **35**: 533–541
Su L-K, Vogelstein B and Kinzler KW (1993) Association of the APC tumour suppressor protein with catenins. *Science* **262**: 1734–1737
Wong MH, Hermiston ML, Syder AJ et al. (1996) Forced expression of the tumour suppressor adenomatous polyposis coli protein induces disordered cell migration in the intestinal epithelium. *Proc Natl Acad Sci USA* **93**: 9588–9593.
Yang VW, Shields JM, Hamilton SR et al (1998) Size-dependent increase in prostanoid levels in adenomas of patients with familial adenomatous polyposis. *Cancer Res* **58**: 1750–1753

 *© 1999 Cancer Research Campaign*
